# Pre-Clinical Study of a Novel Recombinant Botulinum Neurotoxin Derivative Engineered for Improved Safety

**DOI:** 10.1038/srep30429

**Published:** 2016-08-03

**Authors:** Edwin Vazquez-Cintron, Luis Tenezaca, Christopher Angeles, Aurelia Syngkon, Victoria Liublinska, Konstantin Ichtchenko, Philip Band

**Affiliations:** 1 CytoDel^®^ LLC, New York, 10027, United States; 2 New York University School of Medicine, Department of Pharmacology and Molecular Biochemistry, New York, 10016, United States; 3 City College of New York, Department of Biology, New York, 10023, United States; 4 Harvard University, Office of Institutional Research, Cambridge, 02138, United States; 5 Department of Orthopaedic Surgery, New York University Hospital for Joint Diseases, New York, 10003, United States

## Abstract

Cyto-012 is a recombinant derivative of Botulinum neurotoxin Type A (BoNT/A). It primarily differs from *wild type* (*wt*) BoNT/A1 in that it incorporates two amino acid substitutions in the catalytic domain of the light chain (LC) metalloprotease (E_224_ > A and Y_366_ > A), designed to provide a safer clinical profile. Cyto-012 is specifically internalized into rat cortical and hippocampal neurons, and cleaves Synaptosomal-Associated Protein 25 (SNAP-25), the substrate of *wt* BoNT/A, but exhibits slower cleavage kinetics and therefore requires a higher absolute dose to exhibit pharmacologic activity. The pharmacodynamics of Cyto-012 and *wt* BoNT/A have similar onset and duration of action using the Digital Abduction Assay (DAS). Intramuscular LD_50_ values for Cyto-012 and *wt* BoNT/A respectively, were 0.63 ug (95% CI = 0.61, 0.66) and 6.22 pg (95% CI = 5.42, 7.02). ED_50_ values for Cyto-012 and *wt* BoNT/A were respectively, 0.030 ug (95% CI = 0.026, 0.034) and 0.592 pg (95% CI = 0.488, 0.696). The safety margin (intramuscular LD_50_/ED_50_ ratio) for Cyto-012 was found to be improved 2-fold relative to *wt* BoNT/A (p < 0.001). The DAS response to Cyto-012 was diminished when a second injection was administered 32 days after the first. These data suggest that the safety margin of BoNT/A can be improved by modulating their activity towards SNAP-25.

Botulinum neurotoxins (BoNT) are widely used as neuromodulators to treat spastic disorders^1^. They act by inhibiting the release of neurotransmitter in cholinergic neurons, resulting in muscle weakening and paralysis. BoNTs primarily target cholinergic nerves at the neuromuscular junction, inhibiting acetylcholine release and causing peripheral neuromuscular blockade^2^. Clinical indications for use of BoNT products include treatment of dystonias, spasticity disorders, overactive bladder, migraines, and in aesthetic medicine as a treatment for moderate to severe frown lines.

BoNTs have a complex but highly specific trafficking pathway. They are internalized through endocytosis at the presynaptic membrane via an energy-dependent mechanism^3^,^4^, and rapidly appear in vesicles where they are at least partially protected from degradation^5^. The disulfide-bonded BoNT heterodimer of light and heavy chains (LC and HC) interacts with the acidified endocytic vesicle membrane, facilitating translocation of the LC in a fashion similar to the protein conducting/translocating channels of smooth ER, mitochondria, and chloroplasts^6^. Acidification of the endosome is believed to induce conformational changes that allow translocation of the LC to the cytosol upon reduction of the LC-HC disulfide bond^7^. Within the cytosol, the LC displays a zinc-endopeptidase activity, which is specific for each BoNT serotype. BoNT/B, /D, /F, and /G recognize predominantly Vesicle-Associated Membrane Protein-2 (VAMP-2)/synaptobrevin. This integral protein of the synaptic vesicle membrane is cleaved at a single, specific peptide bond that differs for each serotype. BoNT/A, /C1, and /E recognize and cleave SNAP-25, a protein of the presynaptic membrane, likewise at a single, specific peptide bond that differs for each serotype. BoNT/C also cleaves Syntaxin-1, another protein of the nerve plasmalemma^3^. The cleavage of any component of the synaptic release machinery results in inhibition of neurotransmitter release, ultimately leading to neuromuscular paralysis.

The United States Food and Drug Administration (FDA) has approved four pharmaceutical preparations of Botulinum neurotoxins. All of these pharmaceuticals are produced from the natural host, *Clostridium botulinum* (*C. botulinum*). The products differ in their specific potency, the indications for which they are approved, the excipients in the final pharmaceutical composition, and in minor aspects of the composition of the active BoNT pharmaceutical intermediate itself 8. The active ingredient of the pharmaceutical products is either the pure ~150,000 Dalton neurotoxin, or its complex with specific accessory proteins. Notably, because all of the currently available BoNT products are produced from the native microbial source *C. botulinum*, they have not been engineered to modify their pharmacologic properties using the tools of modern molecular biology.

Major adverse events have been reported after therapeutic use of pharmaceutical BoNT products because of off-target actions. All of these products carry a black box warning regarding the potential for systemic intoxication. This toxicity concern limits the use of *wt* BoNT/A for treating disorders involving large muscle groups, which would require high doses of the toxin.

Engineering and purifying recombinant BoNT (rBoNT) that maintain the *wt* toxin’s natural structure and pharmacological activity is challenging because of the protein’s large size and essential disulfide bonding. We have developed modular genetic constructs and an insect cell expression system designed to produce a library of recombinant derivatives of BoNT that retain the structural and trafficking properties of *wt* BoNT^9^,^10^,^11^,^12^. The utilization of an insect cell expression system is also beneficial because it limits the need for using an expression vector or host that could potentially be pathogenic to humans.

In this report, we describe a rBoNT derivative based on the amino acid sequence of *wt* BoNT/A, designated Cyto-012, which is engineered to have an improved safety margin. Cyto-012 has two amino acid substitutions in the catalytic domain of the LC protease (E_224_ > A and Y_366_ > A), which attenuate its catalytic activity and, hence, the molecule’s toxicity. Cyto-012 retains the systemic and intra-neuronal targeting properties of native *wt* BoNT/A. Cyto-012 targets neuromuscular junctions after systemic administration. *In vitro*, the LC of Cyto-012 consistently co-localizes with its natural substrate, SNAP-25, a pre-synaptic cytosolic component of the neuron’s machinery for synaptic vesicle release. Cyto-012 retains some ability to cleave SNAP-25, albeit at a much slower rate than *wt* BoNT/A. Preliminary studies found that when Cyto-012 was injected intramuscularly, it modulated muscle movement and had similar pharmacologic activity to that of *wt* BoNT/A^11^. In this report, we provide proof-of-principle studies demonstrating that the pharmacologic activity of recombinant derivatives of BoNT can be modulated by molecular design to achieve an improved safety margin. The improved safety margin of Cyto-012 is intended to reduce the potential for off-target and systemic adverse events. This can be important when Cyto-012 is used to treat dystonia of large muscle groups, especially for patients with cerebral palsy, post-stroke spasticity and cervical dystonia.

## Results

### Expression, purification, and processing of Cyto-012

**Figure 1:**
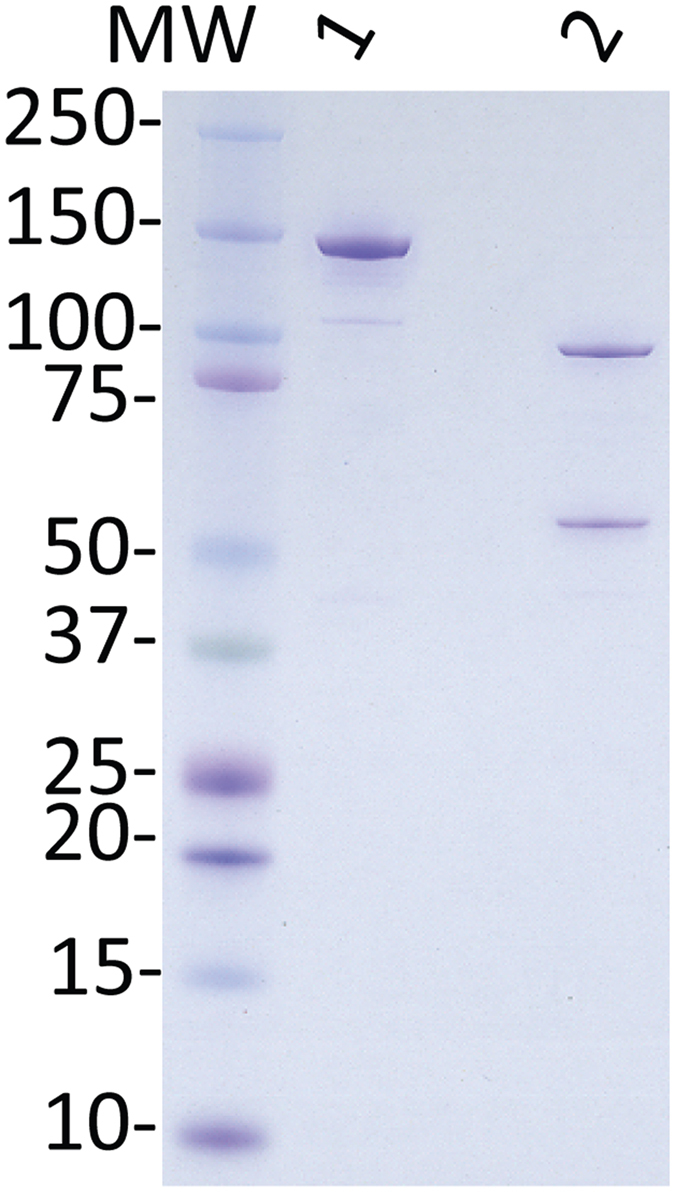
Cyto-012 product analysis. Samples of Cyto-012 (1 μg) were mixed either with non-reducing loading buffer (lane 1) or reducing buffer (2-ME) (lane 2), loaded into a 4–12% acrylamide gel, separated by SDS electrophoresis, and analyzed by Coomassie blue staining.

DNA encoding full-length Cyto-012 was synthesized *de novo*, and optimized for expression in *Sf9* cells. The LC of Cyto-012 has two amino acid substitutions (E_224_ > A and Y_366_ > A), which have been previously shown to reduce the toxicity of rBoNT derivatives by 100,000-fold compared to *wt* BoNT/A^9^. Cyto-012 is recovered from the culture medium as a soluble, stable, disulfide-bonded propeptide. The single chain Cyto-012 precursor propeptide is purified using a 2-step tandem affinity purification procedure with Ni^2+^-NTA agarose and strep-tactin-agarose, utilizing a strep-tactin II tag on the C-terminus and a poly-histidine tag on the N-terminus. A specific protease cleavage site has been placed between the affinity tags at the N- and C-termini, and between the light chain and heavy chain domains of the native *wt* BoNT/A sequence. These cleavage sites enable simultaneous removal of the affinity tags and maturation of the propeptide into the disulfide bonded Cyto-012 heterodimer. After purification to homogeneity, the propeptide is processed with TEV protease to yield the Cyto-012 heterodimer, and the activating protease is removed by affinity chromatography on Ni^2+^-NTA agarose to yield the final active pharmaceutical intermediate, which is polished using gel-filtration chromatography. Protein concentration of the preparation reported here was measured; the final product was sterilized by filtration through 0.22-micron filters, and was shown to be endotoxin-free using the gel clot LAL assay. Purified Cyto-012 analyzed by SDS-PAGE under reducing and non-reducing conditions demonstrates that Cyto-012 has the expected purity, molecular weight, and pattern of disulfide bonding (Fig. 1).

### Cyto-012 co-localizes with, and hydrolyzes its natural substrate SNAP-25

**Figure 2:**
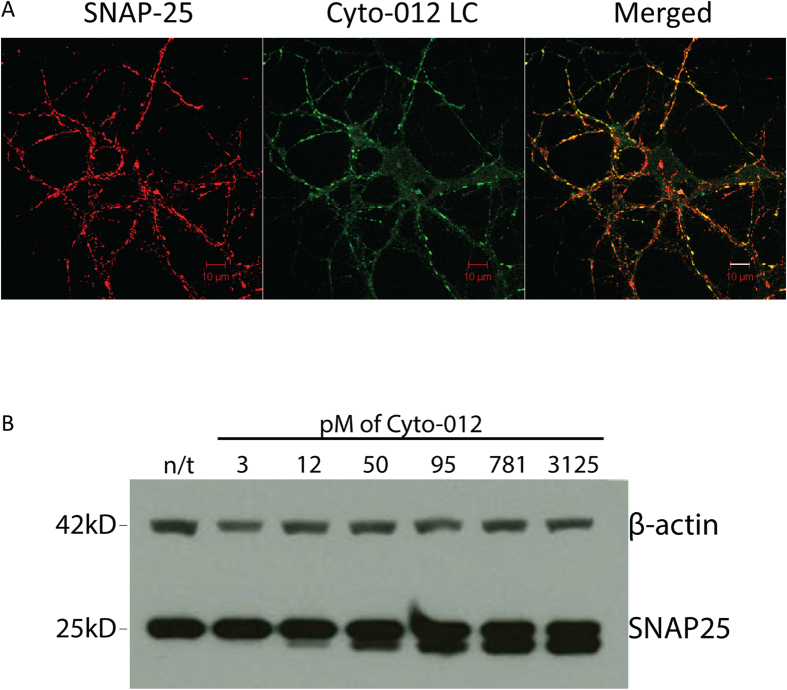
Cyto-012 interacts with its natural substrate SNAP-25. Panel **(A)** Cyto-012 co-localizes with SNAP-25. E19 rat hippocampal neurons were cultured for 14 days and then treated with 25 nM Cyto-012 for 16 hours and prepared for immunocytochemistry as described in materials and methods. Cells were stained for SNAP-25 (red), and Cyto-012 LC (green) using a monoclonal antibody against BoNT/A LC (Clone 5A20). Scale bar 10 μm. Panel **(B)** Cyto-012 cleaves its natural substrate SNAP-25. E19 rat hippocampal neurons were cultured for 21 days and then exposed to different concentrations of Cyto-012 for 48 hours and prepared for Western blot analysis as described in Materials and Methods. Beta-actin was used as a loading control.

To evaluate the intracellular of trafficking of Cyto-012 in neuronal cultures, E19 rat hippocampal neurons were cultured for 14 days and then exposed to 25 nM Cyto-012 for 16 hours. Localization of Cyto-012 and SNAP-25 were analyzed by confocal microscopy. As expected, the LC of Cyto-012 co-localized with SNAP-25 in the presynaptic compartment (Fig. 2A).

To investigate whether Cyto-012 was catalytically active, E19 rat cortical neurons were cultured for 21 days and then exposed to different concentrations of Cyto-012 for 48 hours. The capacity of Cyto-012 to cleave SNAP-25 was analyzed by Western blot. Cyto-012 hydrolyzed SNAP-25 in a dose-dependent manner (Fig. 2B). Beta-actin was used as loading control.

### Intramuscular LD_50_ Analysis of Cyto-012

**Figure 3:**
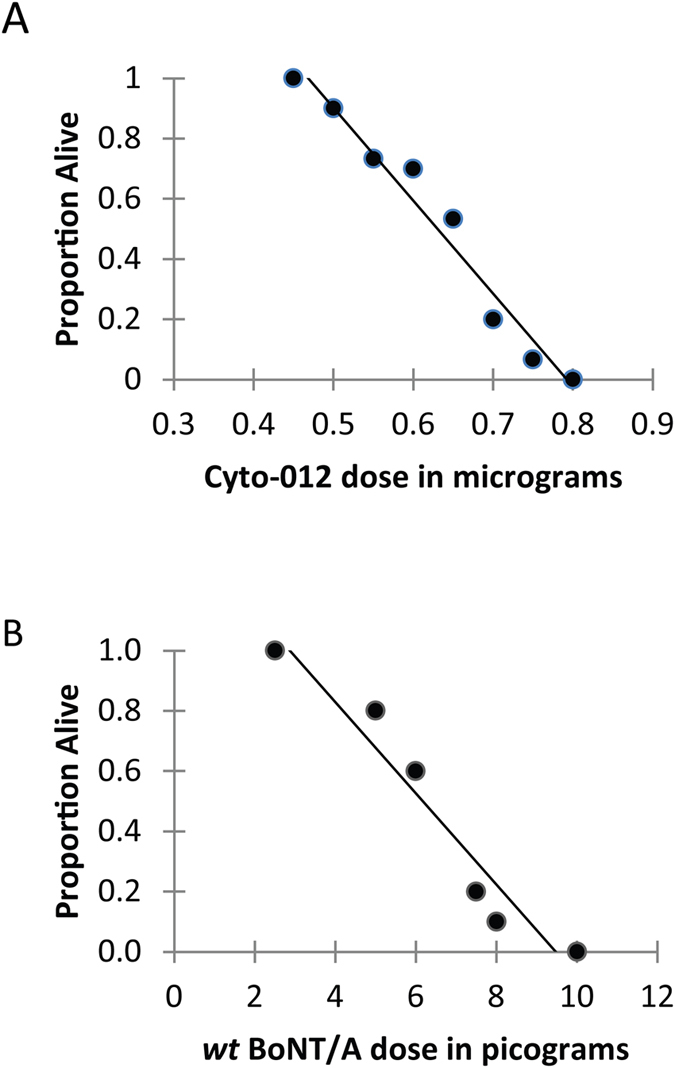
IMLD_50_ analysis of Cyto-012 and *wt* BONT/A. The mouse bioassay assay after intramuscular injection was used to study the lethality (IMLD_50_) of Cyto-012 and *wt* BoNT/A. Plots show the proportion alive at endpoint versus dose of Cyto-012 (micrograms) or *wt* BoNTA (picograms). The IMLD_50_ was calculated by combining data from three independent experiments; *n* = 148 for Cyto-012 and *n* = 148 for *wt* BoNT/A.

The potency of BoNT products is conventionally defined using the mouse lethality assay after systemic intoxication^13^,^14^,^15^. For clinical applications, the potency and toxicity of BoNT products after *im* injections are related, because the lethality observed is typical of BoNT systemic toxemia and likely to be a consequence of diffusion of BoNT away from the site of injection (hind limb muscle) to the systemic circulation. The toxicity of Cyto-012 was defined by mouse lethality analysis in comparison to *wt* BoNT/A following *im* injection in the gastrocnemius muscle. The intramuscular dose lethal to 50% of the mice (IMLD_50_) was determined from a dose-response study (Fig. 3A,B and Table 1). All mice injected with Cyto-012 doses of 0.60 μg or above showed unambiguous clinical symptoms consistent with BoNT-related toxemia, including the development of altered breathing patterns, hind limb paralysis, and wasp-like waist within 24 hours after treatment. The onset of symptoms developed with similar kinetics in mice injected with Cyto-012 or *wt* BoNT/A. All mice injected with Cyto-012 doses of 0.75 μg or higher expired within 48 hours. Similarly, all mice injected with *wt* BoNT/A doses of 12 pg or higher expired within 48 hours. The calculated IMLD_50_ of Cyto-012 was found to be 0.63 μg (95% CI = 0.604–0.656), and the IMLD_50_ of *wt* BoNT/A was 6.22 pg (95% CI = 5.42–7.02) (Table 1). These data show that Cyto-012 is approximately 100,000-fold less toxic than *wt* BoNT/A.

### Pharmacodynamics of Cyto-012

**Figure 4:**
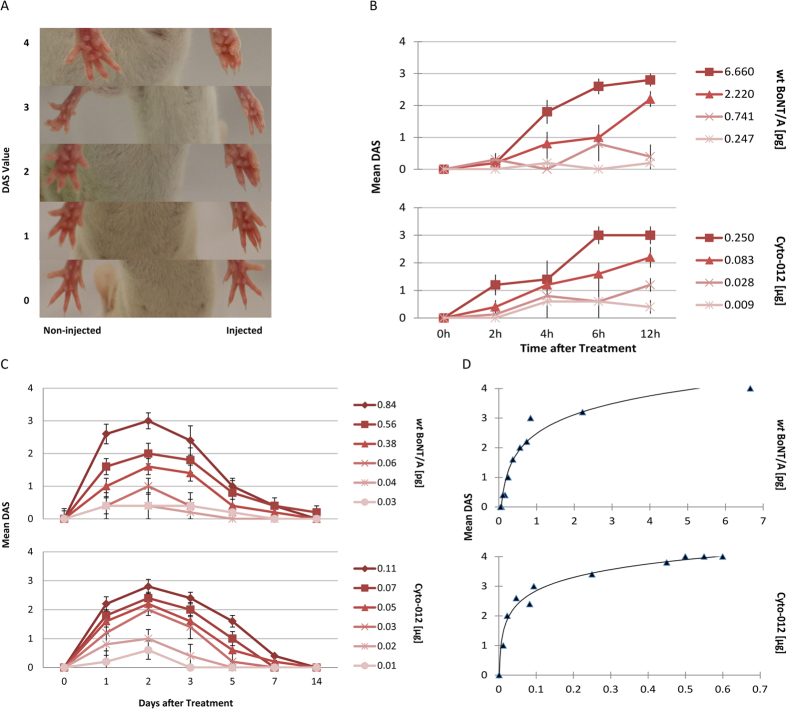
Comparison of pharmacological activities of Cyto-012 and *wt* BoNT/A. Panel **(A)** Representative DAS values on the injected versus non-injected leg. Panel **(B)** Dose-response analysis of onset of muscle paralysis induced by injecting mice with Cyto-012 and *wt* BoNT/A intramuscularly. Plot of the mean DAS versus time (hours) for different concentrations of *wt* BoNT/A (upper panel) and Cyto-012 (lower panel). Panel **(C)** Dose-response analysis of duration of muscle paralysis. Plot of the mean DAS score versus time (days) for *wt* BoNT/A (upper panel) and Cyto-012 (lower panel). Panel **(D)** Plot of the mean DAS response versus dose, at the peak response (Day 2) for Cyto-012 (upper panel) and *wt* BoNT/A (lower panel). The plot represents the combination of three independent experiments; *n* = 90 for Cyto-012 and *n* = 90 for *wt* BoNT/A.

To directly compare the pharmacological properties of Cyto-012 to *wt* BoNT/A, hind limb muscle paralysis and abduction of the hind limbs was assessed in mice given a single *im* injection of Cyto-012 (dose range 0.001 to 0.25 μg) or *wt* BoNT/A (dose range 0.001 to 6.66 pg) in the head of the gastrocnemius muscle. The large difference between the dose ranges examined for Cyto-012 and *wt* BoNT/A was necessitated by the 100,000-fold reduced toxicity of Cyto-012, and its related reduction in potency. The DAS assay and scoring system used to assess the degree of local muscle weakening is illustrated in Fig. 4A.

The onset of muscle weakening was determined by comparing the DAS in the ipsilateral leg to the contralateral leg. The kinetics of onset of DAS values were similar between *wt* BoNT/A and Cyto-012 (Fig. 4B). At higher doses of both Cyto-012 and *wt* BoNT/A, mice exhibited muscle weakening as early as 2 hours post *im* injection.

The peak DAS response for both Cyto-012 and *wt* BoNT/A occurred at day 2, and both slowly and steadily declined over a period of 14 days (Fig. 4C). Duration of muscle weakness induced by either molecule was similar, and exhibited similar dose-response characteristics (Fig. 4B).

Pharmacologic potency of Cyto-012 and *wt* BoNT/A were compared using the DAS assay. Dose response curves for Cyto-012 and *wt* BoNT/A constructed at the peak response (Day 2) after *im* administration were used to calculate the dose giving 50% maximal DAS response (IMED_50_) (Fig. 4D). The IMED_50_ was 0.030 μg (95% CI = 0.026–0.034) for Cyto-012 and 0.592 pg (95% CI = 0.488–0.696) for *wt* BoNT/A (Table 1).

### Comparison of the Safety Margin of Cyto-012 and wt BoNT/A

The safety margin of BoNT can be defined as the ratio between the IMLD_50_ and IMED_50_, where the DAS peak response is used for the calculated IMED_50_^13^,^14^,^15^. The safety margin for Cyto-012 was found to be 21 and the safety margin for *wt* BoNT/A was found to be 10.5 (Table 1). This analysis demonstrates that Cyto-012 has a 2-fold improved safety margin (*p* < 0.001) relative to *wt* BoNT/A.

### Repeat Treatment with Cyto-012

**Figure 5:**
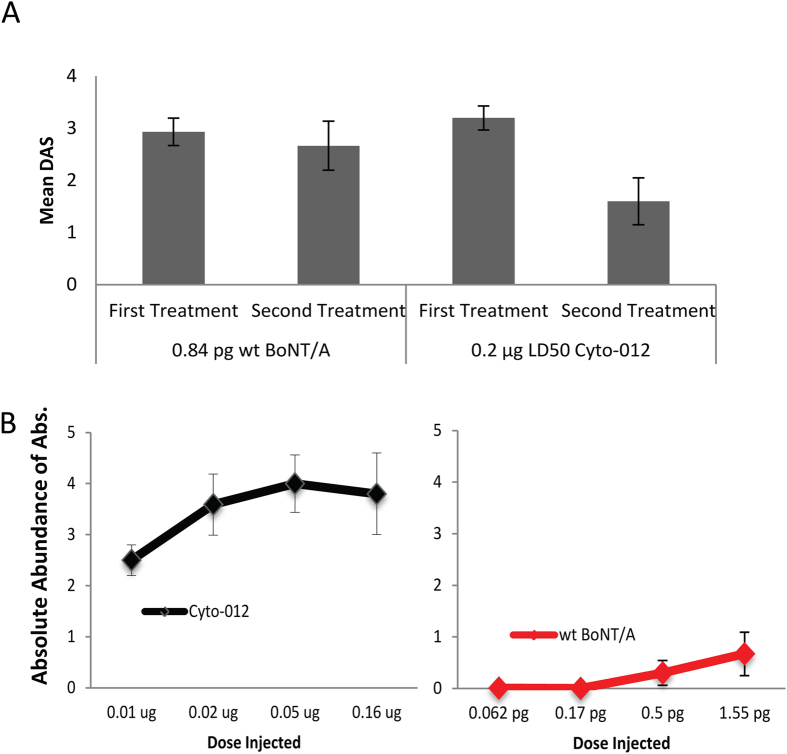
Reduced pharmacological activity of Cyto-012 after repeat treatment is due to increased antibodies titers against BoNT/A. Panel (**A**) Mean DAS in repeat treatment. Mice (*n* = 5) were primed *im* with 0.84 pg of *wt* BoNT/A or 0.2 μg of Cyto-012 on day 0, and challenged with the same dose in the same muscle (ipsilateral leg), 32 days after initial injection. Mean DAS at peak response is presented for both *wt* BoNT/A and Cyto-012 for the first and second treatment. The plot represents the combination of two independent experiments. Panel (**B**) Quantification of antibody titers in serum after repeat treatment of *wt* BoNT/A or Cyto-012. Mice were injected with either *wt* BoNT/A or Cyto-012 on day 0, and day 32 with the same dose and site. Sera was collected 15 days after the second treatment. Plot represents the relative abundance of IgG detected by ELISA, after the second treatment with *wt* BoNT/A (red line) or Cyto-012 (black line), when subtracted to baseline levels.

To determine whether the potency of Cyto-012 was altered during repeated treatment, mice were injected with 0.2 μg of Cyto-012 or 0.84 pg of *wt* BoNT/A on Day 0; then, 32 days after the initial treatment a second injection with the same dose of Cyto-012 or *wt* BoNT/A was administered in the ipsilateral leg. We found that the DAS peak response was diminished after the second treatment with Cyto-012 treated animals, but was unchanged among *wt* BoNT/A treated animals (Fig. 5A).

To investigate whether mice mounted a humoral immune response to Cyto-012, a standard ELISA assay was used to evaluate the presence of antibodies against Cyto-012 in sera collected at days 0 (pre-treatment), 33, and 47 (15 days after the second injection). Mice injected with Cyto-012 at doses between 0.01 to 0.16 μg generated antibodies that reacted against Cyto-012. A similar humoral immune response was not observed in mice injected with *wt* BoNT/A at doses between 0.06 to 1.55 pg (Fig. 5B).

To confirm that mice injected with Cyto-012 developed neutralizing antibodies against Cyto-012, a mouse protection assay was performed. Mice injected with Cyto-012 (range: 0.01 to 0.16 μg) or *wt* BoNT/A (range: 0.06 to 1.55 pg), were challenged with 10 IPLD_50_ units of *wt* BoNT/A 60 days after priming. Mice primed with relatively high doses of Cyto-012 were protected against *wt* BoNT/A challenge; whereas, mice injected with *wt* BoNT/A were not protected, developed symptoms of toxemia and expired within 12 hours.

## Discussion

This is the first report of a recombinant BoNT/A derivative that retains intramuscular pharmacologic activity *in vivo*, despite amino acid substitutions that reduce its systemic toxicity by approximately 100,000-fold. In the current study, Cyto-012 exhibited a meaningful improvement in the safety margin after intramuscular injection in mice compared to *wt* BoNT/A.

Cyto-012 is a full-length, disulfide-bonded, recombinant BoNT/A derivative produced using our previously described expression platform, which has been shown to consistently produce atoxic BoNT derivatives that preserve the systemic and intra-neuronal trafficking properties of *wt* BoNT^9^. Cyto-012 co-localizes with its natural substrate SNAP-25 in primary hippocampal cultures (Fig. 2A), and retains SNAP-25 cleavage activity (Fig. 2B), albeit at a much slower rate than *wt* BoNT/A. Importantly, we found that Cyto-012 has BoNT/A-like pharmacologic activity *in vivo*, as assessed by the standard murine DAS assay as a measure of muscle-paralyzing activity.

We attribute the difference in potency between Cyto-012 and *wt* BoNT/A to the introduced amino acid substitutions, E_224_ > A and Y_366_ > A, in the LC of Cyto-012. Substitution at these amino acid positions by Breidenbach *et al*. was shown to sufficiently attenuate the activity of the BoNT/A light chain protease towards SNAP-25 to enable co-crystallization of LC-SNAP-25 complexes^16^. When E_224_ > Q and Y_366_ > F substitutions were introduced into the LC of BoNT/A, SNAP-25 cleavage activity could no longer be detected in an in-tube assay^16^. The studies performed by Breidenbach *et al*. measured properties of the recombinant isolated LC expression; whereas our studies measured properties of the disulfide-bonded heterodimer. When a recombinant BoNT/A derivative without the amino acid substitutions E_224_ > A and Y_366_ > A was produced using the same expression platform as Cyto-012, it was found to have pharmaceutical potency similar to that of current wt BoNT products^17^. Our findings highlight the importance of expressing the complete heterodimer for studies of the effects of recombinant BoNT/A derivatives on SNAP-25 cleavage in neuronal cultures.

A similar rBoNT/A derivative, BoNT/A atoxic derivative (BoNT/A *ad*), previously produced using the same expression platform and incorporating the same amino acid substitutions, targets the neuromuscular junctions of the diaphragm after systemic administration in mice, and co-localizes with alpha-bungarotoxin^10^. Studies in primary hippocampal neuron cultures demonstrate that: 1) uptake of BoNT/A *ad* is limited to neurons, and the molecule is absent from residual glial cells; 2) BoNT/A *ad* is internalized into a trypsin-protected compartment of neurons and accumulates in the Triton X-100 soluble fraction of the cultures to levels that approach 1 μM; 3) BoNT/A *ad* co-localizes with SNAP-25 and VAMP-2, and displays little co-localization with markers for endosomal compartments^12^. This contrasts markedly with the intra-neuronal trafficking behavior observed for rBoNT derivatives produced using different expression platforms, which find the majority of the rBoNT localized to endosomal compartments, and thus, are unable to reach the cytoplasm^18^. The ability of Cyto-012 to retain pharmacological activity despite its amino acid substitutions further reflects the utility of this expression platform to preserve the native structure and trafficking properties of *wt* BoNT/A.

The studies reported here demonstrate that Cyto-012 has a pharmacologic profile similar to *wt* BoNT/A in terms of the onset and decay of the DAS response (Fig. 4A–D). Because SNAP-25 cleavage activity is attenuated in Cyto-012, higher absolute quantities of Cyto-012 relative to *wt* BoNT/A were required to elicit similar DAS dose response curves. Careful examination of the dose-response curves (Fig. 4C and Table 1) indicate that the improved safety margin arises largely because for Cyto-012, the IMED_50_ dose is 4.8% of the IMLD_50_ dose; whereas, for *wt* BoNT/A, the IMED_50_ dose is 9.5% of the IMLD_50_ dose. For *wt* BoNT/A, the safety margin we report is similar to safety margins reported for pharmaceutical preparations of *wt* BoNT/A (13.1 for BOTOX^®^ and 7.0 for Dysport^® ^13. For Cyto-012, the IMLD_50_ is 21-fold higher than the IMED_50_, providing the improved safety margin we report here.

It is important to consider the possible mechanisms of the improved safety margin we found for Cyto-012. Although it was not surprising that the IMLD_50_ of Cyto-012 was increased approximately 100,000-fold relative to *wt* BoNT/A because of the amino acid substitutions introduced to the LC active site region, it was surprising that Cyto-012 has a significantly improved safety margin, since the change in the LC catalytic activity would be expected to influence IMLD_50_ and IMED_50_ in a similar way. This differential effect on IMLD_50_ and IMED_50_ indicates that Cyto-012 is either more active locally, or is less toxic when it escapes from the site of injection to exert its systemic actions. Multiple mechanisms may come into play. Increased local uptake could result from the higher absolute concentration of Cyto-012 injected relative to *wt* BoNT/A, increasing local neuronal uptake by mass action. It is also possible that local uptake of Cyto-012 is increased because it cleaves SNAP-25 more slowly than *wt* BoNT/A, and is, therefore, less efficient at disabling SV2-mediated uptake of BoNT/A^19^. Another possibility to explain the increased safety margin is the reduced systemic toxicity of Cyto-012. Pharmacokinetic data pertaining to systemic clearance of Cyto-012 relative to *wt* BoNT/A are not yet available; however, the significantly higher concentration of Cyto-012 in the circulation could contribute to accelerated clearance.

Although the safety of current BoNT products is generally considered acceptable, the potential for using the tools of modern molecular biology to engineer recombinant BoNT derivatives for specific clinical improvements has considerable pharmacologic implications. The data presented here demonstrate that the safety margin of BoNT/A can be modified by amino acid substitution. This makes it possible to improve the safety associated with treating dystonia in large muscle groups, where the therapeutic dose comes uncomfortably close to the dose range that has serious systemic side effects, as noted by the Black Box warning in US labeling for BoNT products. This new approach to improving the safety of BoNT products could be especially important for treating children with cerebral palsy, where serious adverse events including death have been reported.

Ongoing work in our laboratories is aimed at developing BoNT/A derivatives that provide an improved safety margin without the increased immunological reactivity observed for Cyto-012. We hypothesize that the engineering of BoNTs with an improved safety margin may be achieved by creating a *de novo* molecule that slightly decreases the rate of SNAP-25 hydrolysis over that of *wt* BoNT/A, with potency high enough to evade priming an immune response.

## Materials and Methods

### Ethics Statement

Experiments involving animals were conducted in accordance with the approval of the City College of City University of New York (CCNY) Institutional Animal Care and Use Committee. CCNY animal facilities are maintained in accordance with the Animal Welfare Act, United States Department of Agriculture Regulations (9 CFR, Parts 1, 2, and 3), and the Guide for the Care and Use of Laboratory Animals (National Academy Press, Revised 2011). CCNY has a currently approved Animal Welfare Assurance Agreement (No. A3733-01) with the NIH Office for Protection from Research Risks.

### Animals

Eight week old, CD-1 female mice (Charles River’s Laboratories) were housed five per cage in a barrier facility, and were maintained on a 12-hour light/dark cycle (7 AM to 7 PM) with *ad libitum* access to food and water. The average weight of mice used for the study was 23.2 grams. Any mice that were above 25 grams or below 21 grams at the time of the first intramuscular injection were not used in the study.

### Botulinum neurotoxin type A and Cyto-012

*Wt* BoNT/A was acquired from Metabiologics, Inc. A recombinant derivative of *Clostridium botulinum* type A (Cyto-012) with two mutations in the core catalytic domain of the light chain was expressed in the select agent laboratory of Dr. Konstantin Ichtchenko at New York University. Cyto-012 was purified to electrophoretic homogeneity and activated by specific protease cleavage, as previously described^9^. Cyto-012 and *wt* BoNT/A were diluted in 1% pyrogen-free mouse albumin (Albumin Biosciences) in 1X DPBS (Invitrogen). Dilutions were made in mouse albumin because albumin is a known protein stabilizer, and stabilizers have been shown to enhance the activity of BoNT/A products at low concentrations in preclinical tests^20^. Endotoxin testing of Cyto-012 was done using the PYROGENT™ Gel Clot LAL Assay (Cat. N19403, Lonza) according to the manufacturer’s instructions. The Genbank accession number for Cyto-012 nucleotide sequence is KU896135.

### Digit Abduction Score Assay

The mouse DAS assay was used to determine the pharmacologic activity of BoNT preparations, measured by muscle weakening effectiveness^13^,^14^,^15^. In the DAS Assay, mice are briefly suspended by their tails to elicit a characteristic startle response in which the animal extends its hind-limbs and abducts its hind digits. This test was used to define the pharmacological activity of Cyto-012 and to compare it to *wt* BoNT/A in mice.

### Intramuscular LD_50_ and ED_50_ studies

Mice were given single unilateral intramuscular injections into the gastrocnemius with the indicated doses. All injections were done using a 2.5 μl Hamilton syringe with 30-gauge needle, using a final volume of 2.5 μl per injection. For IMLD_50_ studies, the mouse lethality assay was used to define potency; death was the primary study outcome measure. Mice were evaluated for up to 5 days after Cyto-012 or *wt* BoNT/A administration, and all symptoms associated with botulism intoxication were recorded. For ED_50_ studies, muscle weakness was assessed using the DAS Assay. DAS was recorded starting at 2 hours post treatment for up to 30 days.

### Preparation and Maintenance of E19 Rat Cortical and Hippocampal Neurons

Timed pregnant Sprague-Dawley rats (Taconic) were used to isolate embryonic-day 19 (E19) cortical and/or hippocampal neurons. Bilateral cortices were dissected from fetal brain, immersed in dissection buffer containing 15 mM HEPES pH 7.2 (Life Technologies), and 0.5% glucose in DPBS without Ca^2+^ and Mg^2+^ (Life Technologies), and were dissociated by incubation in 10 mL of dissection buffer supplemented with 1x Trypsin/EDTA (10x Trypsin/EDTA is 0.5% trypsin/0.2% EDTA, Life Technologies) for 10 minutes at 37 °C. Tissue was triturated using a fire polished Pasteur glass pipette, and cells were counted. The single cell suspension was plated onto poly-L-lysine hydrobromide-coated plates or coverslips in plating medium (1x Minimum Essential Medium-Glutamax^TM^ (Life Technologies), 10% FBS (Fetal Bovine Serum; Life Technologies), 1x Sodium pyruvate (100 mM Sodium pyruvate; Life Technologies), 1x Pen/Strep (Life Technologies). After two hours, plating medium was replaced with maintenance medium (1x Neurobasal medium, Life Technologies), 1x B27 supplement (Life Technologies), and 1x Pen/Strep). Three days after plating, 2 μg/mL cytosine β-D-arabinofuranoside (AraC, Sigma) was added to the maintenance medium to prevent growth of glia. Half of the medium was replaced with fresh maintenance medium every 3 to 5 days.

### Western Blot Analysis

Primary cortical neurons were harvested and solubilized on ice in 200 mL lysis buffer with protease inhibitors (0.5% Triton^TM^ X-100, 100 mM NaCl, 25 mM HEPES, pH 7.5, 10 mM 6-aminocaproic acid, 2 mM benzamidine, 5 mM 4-(2-aminoethyl) benzenesulfonyl fluoride hydrochloride (AEBSF), 2.5 mM EDTA, 325 mM bestatin, 35 mM E-64, 2.5 mM leupeptin, 0.75 mM aprotinin) by passing the sample several times through a 25 gauge needle. Soluble protein lysate was separated from the pellet by centrifuging the samples at 18,000 g at 4 °C for 20 minutes. After lysis, the total protein concentration in each sample was measured, and sample volumes were adjusted with lysis buffer and supplemented with protease inhibitors to equalize concentration. Approximately 30 micrograms of total protein were loaded per lane, separated by reduced SDS PAGE and transferred to a 0.2 mm nitrocellulose membrane (Bio-Rad). Following transfer, membranes were blocked in 10% fat-free milk supplemented with 5% NGS (Normal Goat Serum, Life Technologies) in TBST (150 mM NaCl, 10 mM Tris-HCl pH 8.0, 0.1% Tween^®^ 20) at room temperature for 2 hours. Membranes were incubated with primary antibodies overnight at 4 °C, and with secondary antibodies 45 minutes at room temperature. Primary antibodies used: SNAP-25 (Synaptic Systems, clone 111-011) and beta-actin (Sigma-Aldrich, clone AC-74). Following incubations, blots were washed with TBST 3 times for 5 minutes. Super Signal West Pico chemiluminescent substrate (Thermo Scientific) was used for visualization by autoradiography.

### Confocal Microscopy

E19 rat hippocampal neuronal cultures were maintained *in vitro* for at least 14 days before treatment with Cyto-012. At the end of the treatment, cells were washed with ice-cold DPBS, fixed in 3.7% paraformaldehyde for 15 minutes, and permeabilized in 0.1% Triton X-100 for 5 minutes. Cells were blocked in 10% Normal goat serum for 1 hour, washed, and incubated with primary antibody diluted in 3% NGS, 1X DPBS for 1 hour. Cells were washed with 1X DBPS and exposed to secondary antibody for 1 hour, followed by three washes with DPBS and mounting for analysis. Primary antibodies used included human monoclonal against BoNT/A LC (Clone: 5A20, kindly provided by Dr. James Marks, University of California, San Francisco) and mouse monoclonal antibody against SNAP-25 (Synaptic Systems, clone 111-011). Secondary Antibodies used were goat anti-mouse Alexa Fluor^®^ 555, and goat anti-human Alexa Fluor^®^ 488, both obtained from eBioscience. Image scanning was performed on a Nikon LSM 510 confocal microscope equipped with argon and HeNe lasers producing excitation lines of 488 and 568 nm. Images were analyzed using Zeiss LSM confocal microscopy software (v.4.2).

### ELISA studies

Sera was collected the day before the first injection, 32 days after the initial injection and 15 days after the second injection of Cyto-012 or *wt* BoNT/A. Antigen, single chain Cyto-012, was dissolved in 0.05 M sodium carbonate, pH9.5 to a concentration of 2 μg/mL. 0.05 ml of the antigen (0.1 μg per well) was added to a 96-well plate and incubated for 1 hour at 37 °C. The plates were washed with wash buffer (0.05 M PBS, 0.05% Tween 20, 0.05% BSA). Once antigen was bound, the wells were blocked with 0.2 ml of 2% BSA in wash buffer. The plate was washed briefly, and the sample sera (diluted 1:250, or as otherwise indicated in figure legends or Results) were added and incubated for 1 hour at 37 °C. The plates were washed 5 times and 0.05 ml of anti-mouse HRP conjugated antibody diluted 1:10,000 in 0.1% BSA was added to the wells and incubated for 45 min. The plates were washed 5 times and 0.05 ml of substrate (0.001 M ABTS, 0.05 M sodium citrate pH 4.5, 0.002 M hydrogen peroxide) was added to the wells and allowed to develop for 30 minutes. To stop the reaction, 0.05 ml 10% SDS was added to the wells. To determine the background noise of non-specific binding of antibodies in the sera to the wells, the samples were also tested on plates that did not have an antigen bound to the wells. The plates were analyzed by spectrophotometry readings at 405 nm using molecular devise SpectraMax 190, and Softmax Pro to obtain the absorbance.

### Statistics

Statistical analysis was performed using R software (version 3.1.3) and RStudio version 0.98.1103. IMED_50_ measures were calculated using a linear regression approach to estimate dosage that corresponds to DAS of 2 observed on a second day of the study (considered to be a day of a peak response). IMLD_50_ measures were calculated using a logistic regression approach to estimate dose resulting in death of 50% of animals within 5 days. Confidence intervals were estimated using the delta method^21^,^22^.

## Additional Information

**Accession code:** for Cyto-012 is KU896135.

**How to cite this article**: Vazquez-Cintron, E. *et al*. Pre-Clinical Study of a Novel Recombinant Botulinum Neurotoxin Derivative Engineered for Improved Safety. *Sci. Rep.* **6**, 30429; doi: 10.1038/srep30429 (2016).

## Figures and Tables

**Table 1:** The calculated IMLD_50_, IMED_50_, and safety margin for Cyto-012 and *wt* BoNT/A.

**Sample**	**IMLD**_**50**_	**IMED**_**50**_	SafetyMargin
Cyto-012	0.63 μg(0.604–0.656)	0.03 μg(0.026–0.034)	21
*wt* BoNT/A	6.22 pg(5.420–7.020)	0.592 pg(0.488–0.696)	10.5

The 95% confidential interval is in parenthesis. The values were obtained by combining three independent experiments. The ratio of the safety margin between Cyto-012 and *wt* BoNT/A is estimated to be 2.02 (95% CI = 1.55, 2.58, *p* < 0.001).
